# *PERC*: a suite of software tools for the curation of cryoEM data with application to simulation, modeling and machine learning

**DOI:** 10.1107/S2053230X25007575

**Published:** 2025-09-09

**Authors:** Beatriz Costa-Gomes, Joel Greer, Nikolai Juraschko, James Parkhurst, Jola Mirecka, Marjan Famili, Camila Rangel-Smith, Oliver Strickson, Alan Lowe, Mark Basham, Tom Burnley

**Affiliations:** a The Alan Turing Institute British Library, 96 Euston Road LondonNW1 2DB United Kingdom; b Science and Technology Facilities Council Research Complex at Harwell DidcotOX11 0FA United Kingdom; c Rosalind Franklin Institute, Harwell Science and Innovation Campus, DidcotOX11 0QX, United Kingdom; d University of Oxford OxfordOX1 3QU United Kingdom; e Diamond Light Source (United Kingdom) Harwell Science and Innovation Campus DidcotOX11 0DE United Kingdom; University of York, United Kingdom

**Keywords:** cryoEM, electron cryomicroscopy, Python, *PERC*

## Abstract

Ease of access to data, tools and models expedites scientific research. In structural biology there are now numerous open repositories of experimental and simulated data sets. Being able to easily access and utilize these is crucial to allow researchers to make optimal use of their research effort. The tools presented here are useful for collating existing public cryoEM data sets and/or creating new synthetic cryoEM data sets to aid the development of novel data processing and interpretation algorithms. In recent years, structural biology has seen the development of a multitude of machine-learning-based algorithms to aid numerous steps in the processing and reconstruction of experimental data sets and the use of these approaches has become widespread. Developing such techniques in structural biology requires access to large data sets, which can be cumbersome to curate and unwieldy to make use of. In this paper, we present a suite of Python software packages, which we collectively refer to as *PERC* (*profet*, *EMPIARreader* and *CAKED*). These are designed to reduce the burden which data curation places upon structural biology research. The *protein structure fetcher* (*profet)* package allows users to conveniently download and cleave sequences or structures from the Protein Data Bank or AlphaFold databases. *EMPIARreader* allows lazy loading of Electron Microscopy Public Image Archive data sets in a machine-learning-compatible structure. The *Class Aggregator for Key Electron-microscopy Data* (*CAKED*) package is designed to seamlessly facilitate the training of machine-learning models on electron microscopy data, including electron-cryo-microscopy-specific data augmentation and labeling. These packages may be utilized independently or as building blocks in workflows. All are available in open-source repositories and designed to be easily extensible to facilitate more advanced workflows if required.

## Abstract 2

*PERC* (*profet*, *EMPIARreader* and *CAKED*) is a suite of open-source Python software tools facilitating the curation of cryoEM data utilizing standard data-science libraries.

## 1. Introduction

Cryogenic-sample electron microscopy (cryoEM) is an imaging technique used to obtain the structure of biomolecular objects on near-atomic resolution scales experimentally via transmission electron microscopy (TEM) of cryogenically frozen samples. Due to advancements in hardware and software over the last decade, the resolution achievable via cryoEM reconstruction approaches that possible through X-ray crystallography (Cheng *et al.*, 2015 ▸), with cryoEM being of particular use for determining the structure of macromolecules that are not amenable to other experimental methods such as X-ray crystallography or nuclear magnetic resonance (NMR) spectroscopy (Nogales, 2016 ▸). The images which make up cryoEM data sets commonly have a very low signal-to-noise ratio (SNR) in order to minimize radiation damage. Consequently, the structures of macromolecules are typically obtained by averaging thousands of particle images, which results in large volumes of data per structure.

The development of novel techniques in structural biology depends on the ability to access and utilize data, and this can include synthetic data in some cases (Joosten *et al.*, 2024 ▸; Jeon *et al.*, 2024 ▸). Experimentally reconstructed or predicted atomic models provide a vital tool for accelerating scientific software development in structural biology. Since its founding in 1971, >200 000 experimentally determined macromolecular structures have been deposited in the Protein Data Bank (PDB) archive (Berman *et al.*, 2000 ▸). The availability of this wealth of experimental data has been pivotal in the development of new software in the field, for example *AlphaFold*2 (Jumper *et al.*, 2021 ▸). Atomic models fitted to the 3D maps produced by cryoEM enable researchers to comprehensively analyze and interpret the biomolecule of interest, for example elucidating ligand-binding mechanisms, and allow comparison with related structures, for example revealing differences in conformational or compositional heterogeneity.

Numerous approaches have used cryoEM data sets for the purpose of training deep-learning models. Notable examples have been applied to the cryoEM image-processing and reconstruction pipeline for particle picking (Bepler *et al.*, 2019 ▸; Wagner *et al.*, 2019 ▸), denoising (Bepler *et al.*, 2020 ▸, Buchholz, Jordan *et al.*, 2019 ▸; Buchholz, Krull *et al.*, 2019 ▸), 3D classification and dynamics (Zhong *et al.*, 2021 ▸; Punjabi & Fleet, 2023 ▸; Schwab *et al.*, 2024 ▸) and model building (Jamali *et al.*, 2023 ▸; Si *et al.*, 2020 ▸), among many more examples (Chung *et al.*, 2022 ▸; Donnat *et al.*, 2022 ▸). The Electron Microscopy Public Image Archive (EMPIAR; Iudin *et al.*, 2022 ▸) is a public resource for the raw image data collected by cryoEM experiments and facilitates free access to this data, allowing it to be used for methods development and validation. Many of the resulting algorithms have been widely adopted as they enable quicker processing and/or improved interpretation of the data. There are also packages which allow the simulation of synthetic micrograph data sets, such as *Parakeet* (Parkhurst *et al.*, 2021 ▸) and *MULTEM* (Lobato & Dyck, 2015 ▸), which can be used to optimize data-acquisition strategies (Parkhurst *et al.*, 2024 ▸) and provide ground-truth information to allow a greater understanding of the functioning of data-processing algorithms (Joosten *et al.*, 2024 ▸).

Being able to easily access these open repositories of experimental and simulated data is crucial for accelerating scientific software development in structural biology. However, in practice doing this can be challenging, particularly for nondomain specialists or those new to the field. Each atomic model database has its own manual download system or individual Python package, and external software packages, such as *BioPython* (Cock *et al.*, 2009 ▸) or *ProDy* (Bakan *et al.*, 2011 ▸), typically only focus on giving access to a single database, such as the PDB. On the other hand, deep-learning-based approaches require large amounts of data to train the algorithms. For example, EMPIAR data sets can have hundreds of files and sizes of the order of terabytes, meaning that downloading and managing these data sets can become a barrier to the development of deep-learning methods. Additionally, the currently recommended tools to download data from EMPIAR either use proprietary software, require a user account or necessitate a web browser. As a result, data handling and management can become a largely manual task, limiting the ease of algorithm development, particularly for researchers new to the field.

**Figure 1:**
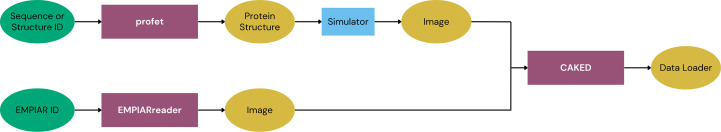
Flowchart of the overall pipeline utilizing *profet*, *EMPIARreader* and *CAKED*. Data can be retrieved from the preferred source with *profet* (and then, for example, input into a simulator to achieve a synthetic image populated with the structure) and *EMPIARreader* allows lazy loading of the data from the specified directory; further processing can then be performed with *CAKED*, which will collate the data ready to feed to a machine-learning model. The green ovals correspond to the inputs, purple rectangles to the implemented packages, the blue rectangle to the outside sources and yellow ovals to the output.

In this paper, we present a suite of software packages which we have developed in order to address these issues: *profet*, *EMPIARreader* and *CAKED*. These packages may be used independently or utilized to build workflows, such as that shown in Fig. 1 ▸. With *profet*, users can conveniently download individual sequences directly using Python by simply specifying their UniProt ID (The UniProt Consortium, 2022 ▸). Users can specify which database they would like to use by default and, if the structure is available from that source, it will be downloaded. If the structure is not available from that source, *profet* will seek to download it from an alternative database (*i.e.* if the requested source is the AlphaFold database and the structure is not available, it then retrieves it from PDB). *EMPIARreader* is an open-source tool which provides a Python library to allow lazy loading of EMPIAR data sets into a machine-learning-compatible format. Lazy loading ensures that data are not loaded into memory until the moment that they need to be accessed, which allows the management of large data sets and is particularly suitable for machine learning. *EMPIARreader* additionally provides a simple, lightweight command-line interface (CLI), which allows users to search and download EMPIAR entries using glob patterns or regular expressions and then download files via FTP or HTTP(S). With all of the data accessed and downloaded, it is still necessary to load them into a machine-learning readable format. In addition to EM experiments, the EMPIAR database is also home to numerous tomography (cryoET) experiments, for which there are currently fewer machine-learning applications. Easy access to cryoET data could facilitate a future increase in the use of machine learning for tomography. For PyTorch, we have developed the *Class Aggregator for Key Electron-microscopy Data* (*CAKED*) software package. *CAKED* loads the images (both for single-particle images or movies, and tomographic tilt series) from a local source. After loading and augmentation, the data are stored in a PyTorch DataLoader class ready to be used for training/classification. *CAKED* is designed to allow easy incorporation of different databases and is designed to allow future extension to facilitate seamless loading directly from the online data sources without local storage. This allows seamless workflows (see Fig. 1 ▸) where ML practitioners can access and utilize the data, whether from EMPIAR, *Parakeet* or elsewhere, for training or deploying ML models. The software packages are open source and available to download from their GitHub repositories.

## 2. Software packages

While the software packages can be used in the workflow exemplified in Fig. 1 ▸, each package can be used individually for their defined purposes.

### 2.1. profet

**Figure 2:**
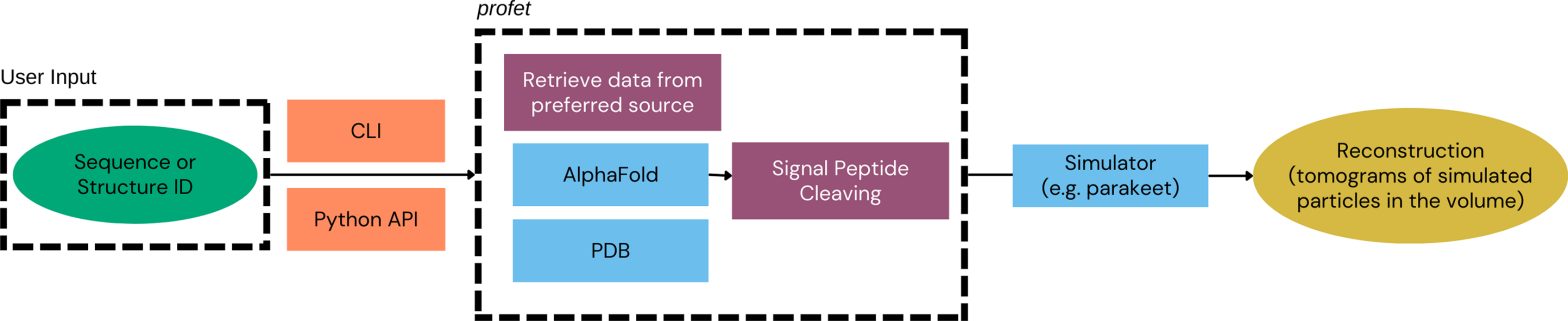
Flowchart of the *profet* pipeline. The green oval corresponds to the input, orange rectangles to the access type, purple rectangles to the implemented packages, blue rectangles to outside sources and the yellow oval to the output. The user can input a protein-structure or protein-sequence ID and *profet* will provide the matching output data from the selected source after optional signal-peptide cleavage for further processing or utilization by the user.

The *profet* library provides a convenient unified interface to retrieve structures of biological macromolecules from either the PDB or AlphaFold database simply by specifying the UniProt ID (Fig. 2 ▸; a PDB ID is also accepted). When a structure file is downloaded, it is cached to a local directory; if the same structure is requested again, either during the same session or a later session, then the cached structure file will be used, preventing redundant downloads. If there are multiple structures associated with the same UniProt ID, the first entry is used. Various potential applications require the ability to download many structures on demand, including protein-matching algorithms for visual proteomics (Wagner *et al.*, 2019 ▸; Mirecka *et al.*, 2022 ▸; Famili *et al.*, 2025 ▸), large-scale models in molecular-dynamics simulations (McGuffee & Elcock, 2010 ▸; Stevens *et al.*, 2023 ▸) and electron-microscopy simulations (Parkhurst *et al.*, 2021 ▸). In addition to a straightforward Python API, *profet* provides a simple command-line interface, enabling the user to utilize *profet* either as part of a script or as a standalone program.

Commonly, structures are downloaded directly from the respective portal interfaces. For batch downloads, it is necessary to have scripting skills (RCSB PDB, 2024 ▸). However, with computational power increasing and facilitating the use of large training sets, it is becoming increasingly important to automate the pipeline of PDB structure access from both experimental and simulated sources, which *profet* provides a portal to. As an added feature, *profet* provides the option to cleave the signal peptides from the retrieved structures (as commonly found, for example, in *AlphaFold* structures). It also offers the deletion of hydrogens, waters or heteroatoms. Furthermore, *profet* is scalable in that it has the ability to add other databases as search options, such as CATH (Sillitoe *et al.*, 2020 ▸) for example, by providing a template for accessing database APIs which can be found on the *profet* GitHub page under profet/template/database.py. Example use can be found in the profet.ipynb Jupyter notebook available in the package repository.

### 2.2. EMPIARreader

Raw cryoEM image data sets can be deposited into the online public image archive EMPIAR (Iudin *et al.*, 2022 ▸). There is a loose schema to follow, but generally each deposited data set is structured according to the needs or preferences of the depositing user, with no particular directory tree enforced. With over 2040 entries and >4.3 petabytes (PB) of data hosted (as of October 2024), EMPIAR has become an important resource for the structural biology community, amassing over 700 citations in published works.

In addition to conventional reuse, such as to compare new software with popular entries, data sets from EMPIAR have been used extensively for training and validating cryoEM-related deep-learning algorithms, particularly those which rely on raw image data. To make optimal use of the archive it is essential that the data sets are easily obtainable, and their size does not hinder accessibility or algorithm performance. The current recommended methods to download data from EMPIAR are via (i) the IBM Aspera Connect web interface (IBM, 2023 ▸), (ii) the IBM Aspera CLI (IBM, 2020 ▸), (iii) Globus (Foster, 2011 ▸; Allen *et al.*, 2012 ▸) or (iv) HTTP(S) or FTP from the entry web page using an internet browser (Iudin *et al.*, 2022 ▸).

**Figure 3:**
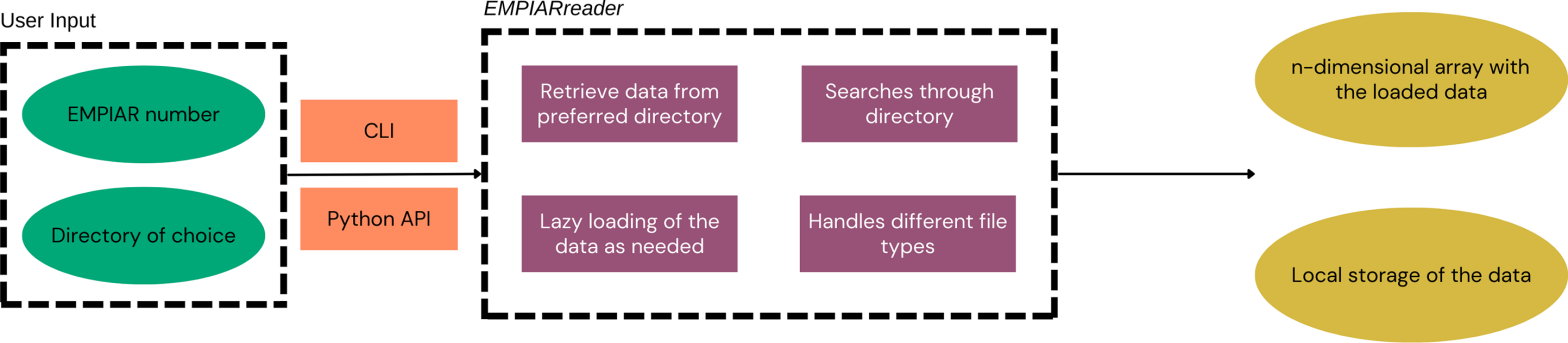
Flowchart of the *EMPIARreader* pipeline. The green ovals correspond to the inputs, orange rectangles to the access type, purple rectangles to the implemented packages and yellow ovals to the output. *EMPIARreader* allows data and metadata to be downloaded in a dynamic manner through lazy loading, while also providing a simple interface for downloading EMPIAR files persistently to disk if required.

These methods all require that data are downloaded and persisted before use and offer limited configurability and automation in data selection and access. To address this and to provide a way to integrate EMPIAR data into machine-learning codebases, we have developed *EMPIARreader*, an open-source tool which provides a Python library to allow lazy loading of EMPIAR data sets into a machine-learning-compatible format (Fig. 3 ▸). It parses EMPIAR metadata, uses the mrcfile library (Burnley *et al.*, 2017 ▸) to interpret MRC files, supports common image-file formats and uses the starfile library (Burt, 2020 ▸) to interpret STAR files. *EMPIARreader* additionally provides a simple, lightweight CLI, which allows users to search and download EMPIAR entries using glob patterns or regular expressions and then download files via FTP or HTTP(S).

Therefore, in contrast to other methods, *EMPIARreader* allows data and metadata to be downloaded in a dynamic manner through lazy loading, whilst also providing a simple interface for downloading EMPIAR files persistently to disk if required. *EMPIARreader* allows the granularity of downloads to be configured from an entire EMPIAR entry down to individual files. This makes *EMPIARreader* flexible enough to handle tasks from downloading a single file to downloading custom subsets of data from different EMPIAR entries. In principle, *EMPIARreader* allows any user to make use of the entire data archive without utilizing local disk-storage resources. It is envisioned that this utility will be particularly useful for the training of ML models and allow improved algorithmic performance by allowing fast and lightweight access to diverse training data. To see examples of the *EMPIARreader* API and CLI please refer to Section 3.2 or the Jupyter Notebook (https://github.com/ccpem/empiarreader/blob/main/examples/run_empiarreader.ipynb) accessible on the *EMPIARreader* GitHub page. *EMPIARreader* documentation can otherwise be found at https://empiarreader.readthedocs.io/en/latest/.

### 2.3. CAKED

**Figure 4:**
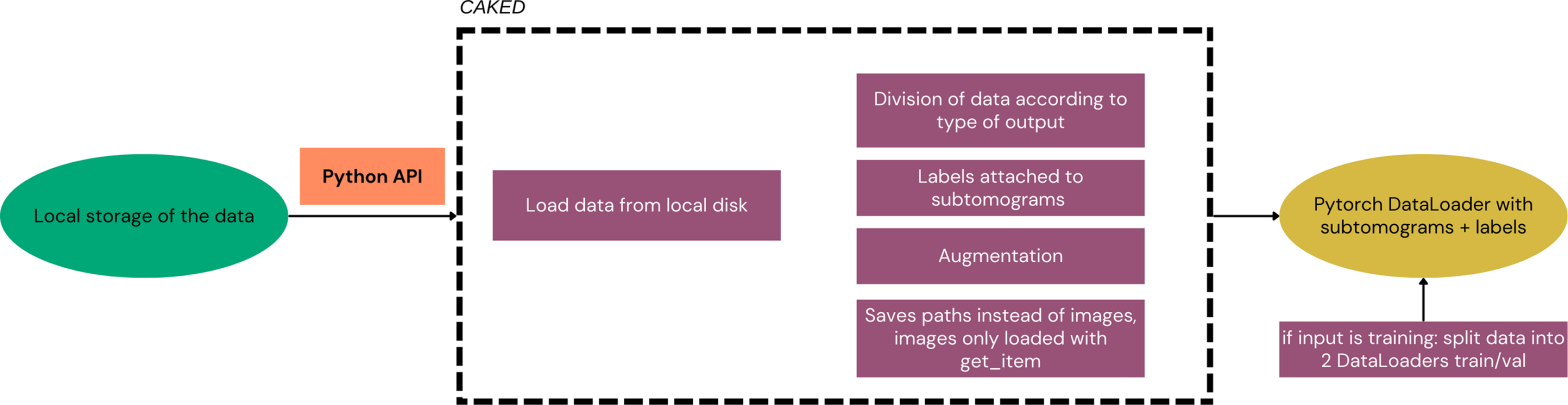
Flowchart of the *CAKED* pipeline. The green oval corresponds to the inputs, the orange rectangle to the access type, purple rectangles to the implemented packages and the yellow oval to the output. *CAKED* is a software package that decouples data loading and processing from the analysis, and represents the data in a format suitable for machine learning. *CAKED* extends the standard PyTorch Dataset and DataLoader primitives, and is itself extendable for more specific data sets and applications.

*CAKED* is a software package that decouples data loading and processing from the analysis (Fig. 4 ▸). The package extends the standard PyTorch (Ansel *et al.*, 2024 ▸) Dataset and DataLoader primitives, and is itself extendable for more specific data sets and applications. After downloading cryoEM data, either to a local storage or in cache, it is necessary to represent it in a format suitable for machine learning, including both image files (which include their own metadata) and metadata files (such as picked-particle metadata files).

*CAKED* is capable of processing both 2D and 3D image data in both MRC (Cheng *et al.*, 2015 ▸) and Numpy (Harris *et al.*, 2020 ▸) file formats. It assumes a file-naming convention containing a class, followed by an underscore, followed by any other useful suffix information. As an extension of the PyTorch Dataset class, it makes use of various existing PyTorch transforms as well as implementing a number of its own cryoEM/cryoET-specific preprocessing functions, including rescales, smoothing/blurring and various normalization methods. Furthermore, it is compatible with any other existing transforms available in torchvision (Marcel & Rodriguez, 2010 ▸). The data is then wrapped in a PyTorch DataLoader class producing an iterable, along with labels and associated information related to each data point (*e.g.* filename suffix). The data is also automatically split into training and validation sets, as per the requested percentage split.

As the package is built in order to be scaled for large data sets, *CAKED* stores the paths instead of the data itself: this helps with the deployment of large data sets as each image is only accessed whenever the get_item function is called. For larger files, support for memmap objects or dask arrays will be added in future. While currently the methods implemented consider only the local storage, a template for other sources (for example, from cache) and the incorporation of different databases is available. Furthermore, this template could be extended for on-the-fly loading directly from the online databases bypassing the local storage in the future (*e.g.* by calling *EMPIARreader* and *profet* directly). Example usage can be found in the notebook available in the GitHub repository (https://github.com/ccpem/caked/blob/main/perc.ipynb). It includes an example of a workflow joining all packages together.

## 3. Applications

### 3.1. *profet* applications

#### 3.1.1. Installation

Install *profet* using pip:
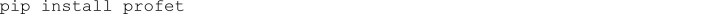


To install the development version, which contains the latest features and fixes, installation can be directly performed from GitHub using
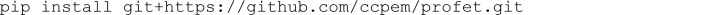


To test the installation, you need to have the *pytest* and *pytest-cov* packages installed, which can be performed as follows:
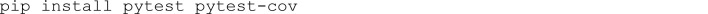


Then navigate to the root directory of the package and run *pytest*. This code has been designed and tested for Python 3.

#### 3.1.2. Downloading PDB or AlphaFold database entries from Python

The *profet* library has a high-level Python API that can be used to download entries from both the PDB and AlphaFold database through a single unified object-oriented interface. An example of how to access this functionality through the main protein fetcher class, profet.Fetcher, is shown in the following code snippet:
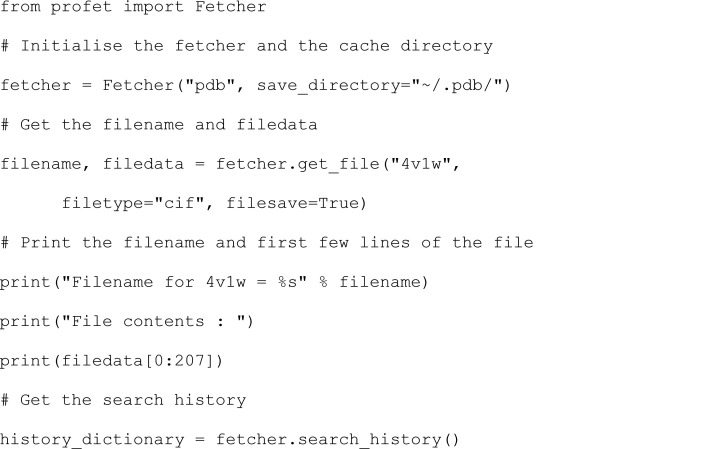


This results in the following output:
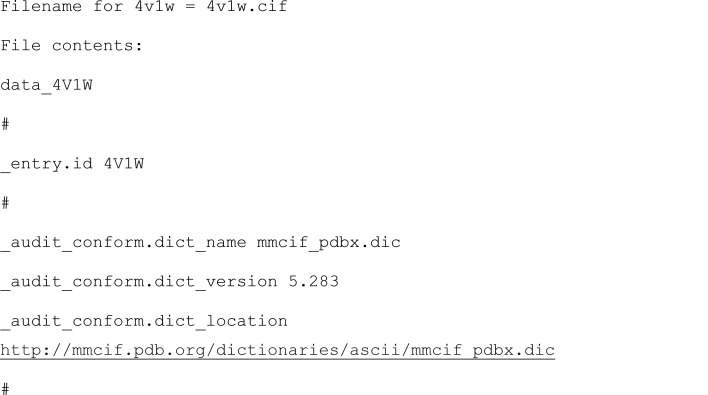


During initialization, the default database can be specified in the constructor of the profet.Fetcher class. This should be a string containing either ‘pdb’ for the PDB or ‘alphafold’ for the AlphaFold database. In the example, the PDB was specified. The directory to use for caching structure files can also be specified at this point by setting the save_directory keyword in the constructor. After initialization, the fetcher can then be used to download structures from the specified database. This can be performed by using the profet.Fetcher.get_file method and by specifying the ID of the protein of interest. In the example, an apoferritin model (‘4v1w’) is downloaded using the PDB ID.

The universal identification value that works across platforms is the UniProt ID. This is due to *AlphaFold* categorizing structures only by their unique UniProt ID, while the PDB has a corresponding ID, as the same molecule can have different experimental entries. If the entry does not exist in any of the databases, then an exception is raised.

When downloading the protein structure, the filetype keyword can also be specified to choose between ‘cif’ or ‘pdb’ file if available. If the requested file type is not available then *profet* will attempt to download whichever file type is available. For example, if ‘cif’ is requested but only ‘pdb’ is available, the PDB file will be downloaded. If no file type is specified then the cif file will be download if present, otherwise the PDB file will be downloaded. Additionally, the filesave boolean flag can be used to specify whether or not the protein structure should be saved automatically to disk. By default this is set to False, in which case the returned filename is None and only the filedata is returned as a binary string. If filesave is True, then the file is saved into the current working directory. Finally, the profet.Fetcher.search_history function can be used to access the list of previously searched structures. The command will show a dictionary of the IDs searched by the fetcher and the databases where they are available as follows.


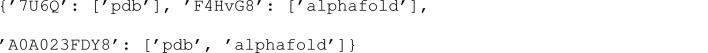


The functionality can be tested using the run profet.ipynb Jupyter notebook available in the package repository.

#### 3.1.3. Remove signal peptides and other functionalities

Once a structure is downloaded using get_file, the function remove from the Fetcher class lets you make a selection of things to remove from the structure file, which is often useful for simulation work. The option signal_peptides compares the sequence of the structure to the UniProt database for any signal peptides included in the structure. It then automatically deletes the signal peptides from the structure. Similarly, H atoms, water molecules or heteroatoms can all be selected to be deleted from the structure. If no output name is given, the processed structure is saved as a separate file, with the options added to the filename (*i.e.* the deleted residue positions, or ‘nohydrogens’).


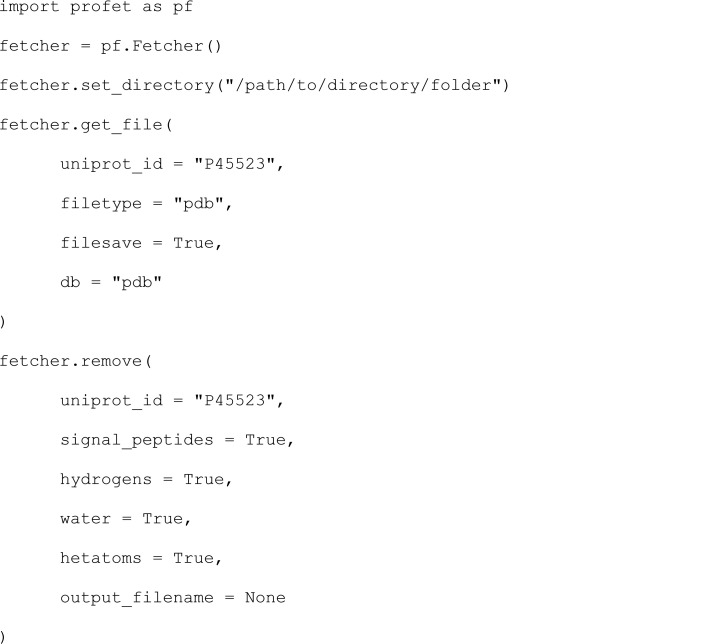


This will save p45523 1q6u.pdb and p45523 1q6u nosignal1to25 nohydrogens nowater nohetatm.pdb to the specified directory.

#### 3.1.4. Downloading PDB or AlphaFold entries from the command line

The *profet* library also has a CLI that mirrors the Python API and which can be used to download entries from both the PDB and AlphaFold database. An example of how to use the *profet* command-line program is shown in the following code snippet.


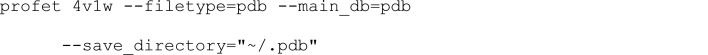


In this example, the entry ‘4v1w’ is to be downloaded from the PDB as a .pdb file. The file will be cached in the hidden home cache directory for future use (defaults to ~/.cache/pdb/).

### 3.2. *EMPIARreader* applications

#### 3.2.1. Installation

*EMPIARReader* can be installed as a PyPi package using Python version ≥3.8 via


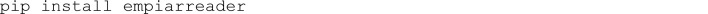


Otherwise, installation can be performed with


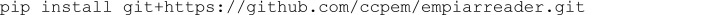


#### 3.2.2. Using the *EMPIARreader* Python interface

For this example, we open EMPIAR entry 10943, investigate the associated metadata and load an image data set.


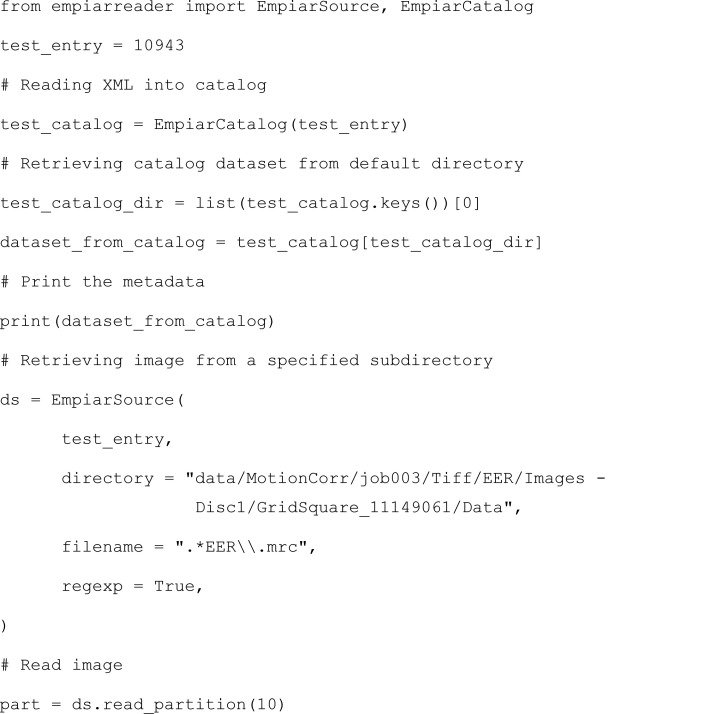


Every EMPIAR entry has an associated XML file which contains the metadata pertaining to one or more image data sets present in the entry. These metadata can be accessed by loading the entry into an EmpiarCatalog. To retrieve the metadata for a given image data set from the catalog, one needs to specify which image data set to load. In the case above, there is only one so we choose the key in position 0. However, the file(s) that a user may wish to download are not always present in one of the directories defined as an image data set in the XML. In this case, one can specify the directory from which they would like to retrieve the images and *EMPIARreader* can load the data set from an EmpiarSource, using the EMPIAR entry number and the directory of the images. In the above case, we also specify that we want the MRC files from the specified directory. The data set is loaded lazily using Dask (Rocklin, 2015 ▸), so the images are loaded one at a time when ‘read partition’ is called. To choose an image, one can just pick the corresponding partition; for example, partition 10 for the 11th image. This example can be visualized in the Jupyter Notebook provided in the *EMPIARreader* repository.

#### 3.2.3. Using the *EMPIARreader* command-line interface

You can use the *EMPIARreader* CLI to search the EMPIAR archive one directory at a time to find what you are looking for before then downloading those files to disk. First, you will need to choose an EMPIAR entry; in this example EMPIAR entry 10934 is used. Here, we use a glob wildcard (--select "*") to list every subdirectory and file in a readable format:


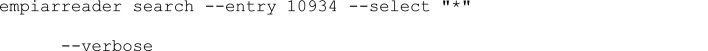


which returns


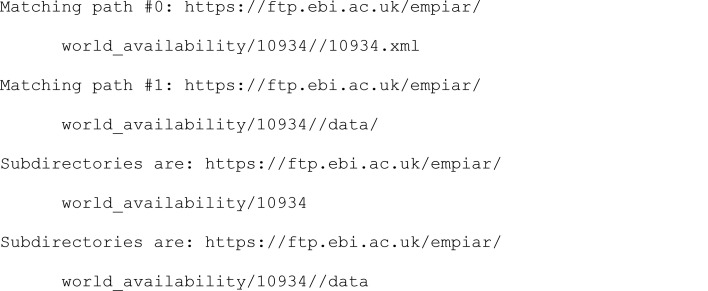


We have found the XML containing the metadata for the entry and a subdirectory called ‘data’. To look inside you can add the --dir argument and repeat recursively until you find the directory you are interested in:


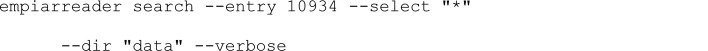


Once you have found one or more files which you want to download from a directory in the EMPIAR archive you can create a list of URLs using the --save search argument:


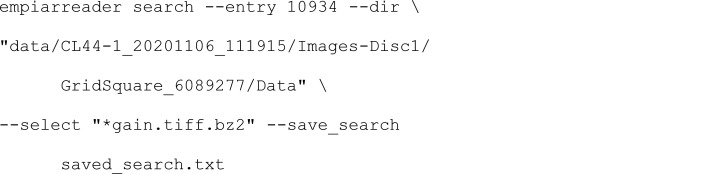


Using the workflow described above, a user can quickly search and identify data sets that fulfill their criteria. These can then be downloaded using the download utility of the CLI. A user simply needs to specify the file list and a directory to download the files into:


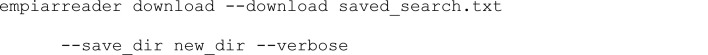


### 3.3. *CAKED* applications

#### 3.3.1. Installation

*CAKED* can be installed via the PyPi package:


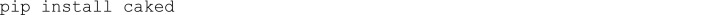


To install the development version, which contains the latest features and fixes, installation can be directly performed from GitHub using


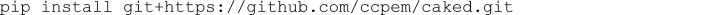


or by cloning the latest version on GitHub:


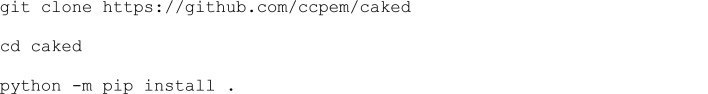


#### 3.3.2. Local loading of data

In this section, we will demonstrate the *CAKED* application considering the local storage of a data set. Each image is stored with the respective class as the prefix of the filename (as of now this has to be performed manually, but in future work where *EMPIARreader* and *profet* will be incorporated to *CAKED* for on-the-fly processing, this will be performed automatically). The first step is to create a DiskDataLoader object. Any required arguments are passed to the object during instantiation and are all optional: the pipeline from which to load (during the time of writing, only "disk" is available), the list of classes in the data set, size of the data set (optional to limit the data for debugging), whether the dataloader is used for training (default is True), the list of transforms to apply to the data and, finally, whether to save to disk the data after processing or not.


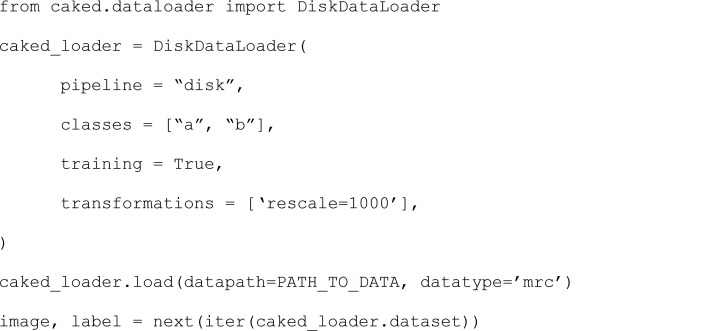


After the loader has been instantiated, the data can be loaded using the load function. In this case, the path to the data must be provided, as well as the datatype (the default is mrc). When the list of transformations in the loader is not empty, the process function is called in order to apply the transformations to the data. Finally, images can be accessed with iterator functions such as next() and iter(). Currently the iterator is expected to return two values: data and label. However, one can easily extend this by overwriting the caked.dataloader.DiskDataset class. An example of this can be found in Section 2 of the Jupyter notebook available in the *CAKED* GitHub repository.

## 4. Discussion

The democratization of cryoEM data into a machine-learning-compatible pipeline is an important step in the automation of processes to further analyze the data. In this paper, we present three packages that can be used to deal with manual bottlenecks in the data-curation process. With *profet*, users can now download protein structures from experimental data and feed them straight into their simulation or modeling pipelines. *EMPIARreader* enables the lazy loading of large data sets downloaded from the online archive onto a Python format such as a numpy array, or direct download to local storage. Finally, *CAKED* can load different types of images into a PyTorch DataLoader, which can be directly used with PyTorch-compatible models. Importantly, all three of these packages can be used as part of the data flow within a single software pipeline, which needs, for example, both experimental and simulated data (for example from *Parakeet*) injected into a model (for example for generative model studies). Furthermore, the seamless integration of all three packages can be performed within a Python workflow, which is demonstrated in a Jupyter notebook in the *CAKED* GitHub repository (https://github.com/ccpem/caked/blob/main/perc.ipynb). Further developments are also possible given the modularity of the tools: *profet* has a template to easily integrate other databases (for example, CathDB; Sillitoe *et al.*, 2020 ▸), *EMPIARreader* can be an off-the-shelf Python API tool for EMPIAR, and *CAKED* will be extended to enable on-the-fly loading without disk storage via *EMPIARreader* and *profet*. All of the source code is open source (*EMPIARreader*, BSD 3-Clause ‘New’ or ‘Revised’ License; *profet* and *CAKED*, MIT License) and development is performed collaboratively, therefore it is possible for users of the packages to add other sources of data, and external contributions to the projects are welcome (see the contribution pages in each repository: *profet*, *EMPIARreader*, *CAKED*). It is our hope that these tools will simplify the process of accessing data and training ML models or performing any other computational analysis and reduce the setup cost for researchers in the field.
